# Comparison of Surgical Stress Responses During Spinal and General Anesthesia in Curettage Surgery

**DOI:** 10.5812/aapm.20554

**Published:** 2014-08-13

**Authors:** Fereshteh Amiri, Ali Ghomeishi, Seyed Mohammad Mehdi Aslani, Sholeh Nesioonpour, Sara Adarvishi

**Affiliations:** 1 Pain Research Center, Ahvaz Jundishapur University of Medical Sciences, Ahvaz, Iran; 2 Department of Anesthesiology, Ahvaz Jundishapur University of Medical Sciences, Ahvaz, Iran; 3 Student Research Committee, Nursing and Midwifery School, Ahvaz Jundishapur University of Medical Sciences, Ahvaz, Iran

**Keywords:** Anesthesia, Blood glucose, Curettage, Spinal

## Abstract

**Background:**

Response to the surgical stress is an involuntary response to metabolic, autonomic as well as hormonal changes that leads to heart rate and blood pressure fluctuations.

**Objectives:**

This study aimed to investigate the effect of general versus spinal anesthesia on blood sugar level and hemodynamic changes in patients undergoing curettage surgery.

**Patients and Methods:**

In this randomized clinical trial, 50 patients who were candidate for elective curettage surgery were divided into two groups of general (n = 25) and spinal (n = 25) anesthesia. In both groups, blood glucose level was evaluated 10 minutes before, 20 and 60 minutes after initiation of anesthesia. Also, heart rate and mean arterial blood pressure were evaluated at 10 minutes before, 10, 20, 30, 40, 50 and 60 minutes after intiation of anesthesia.

**Results:**

There was not significantly difference between blood glucose level of both groups during 10 minutes before, 20 and 60 minutes after the intiation of anesthesia. Heart rate changes in the general and spinal groups compared to the baseline level were decreased up to maximum12.5% and 14.5%, respectively. The mean arterial pressure changes in the general and spinal groups compared to the baseline level were decreased up to maximum 5.4% and 8%, respectively.

**Conclusions:**

Blood glucose and hemodynamic changes caused by surgical stress were not significantly different between two groups.

## 1. Background

The severity of stress during surgery affects not only patient outcomes but also health care system (1). Type of surgery has an important role on the surgical stress rate. Also, women experience more surgical stress than men, resulting in hemodynamic fluctuation (2).

Through physiological responses (endocrine) and psychological stress (anxiety and fear) of anesthesia and surgery increase secretion of cross-regulating hormones (catecholamines, cortisol, glucagon and growth hormone) and resulting in plasma proteins augmentation, sodium retention, potassium loss, and increase of blood glucose level. The increased sympathetic activity and noradrenaline levels caused by surgical stress not only lead to decrease of insulin secretion, glucose consumption as well as gluconeogenesis augmentation, but also resulting in hyperglycemia (3), increasing in postoperative infection and mortality due to immunity system weakness. Moreover, these hemodynamic changes lead to neural, renal and cardiovascular damage (4).

Activation of the sympathetic nervous system, increase of catabolic hormone release and pituitary gland suppression are considered as a response to surgical stress, in clinical practice these activities cause changes in heart rate, blood pressure and biochemical fluctuations of noradrenaline, adrenaline, dopamine, and cortisol (5). Above all, these fluctuations prolong hospitalization and delay patients discharge (6).

In general, there are three main methods for balancing responses to stress during surgery including neural blockade by epidural or spinal anesthesia, which inhibit transmission of impulses from the site of trauma-intravenous administration of high-dose of strong opioid analgesics-which block hypothalamic pituitary gland function and infusion of anabolic hormones such as insulin that causes changes in the hormonal status of the patient (7).

Anesthesia techniques, which can reduce surgical stress and consequently hyperglycemic responses, considered as regional, neuraxial and general anesthesia (8).

## 2. Objectives

Regarding to different kinds of anesthesia techniques and surgery, and due to that women experience more surgical stress than men, the present study aimed to evaluate the effects of spinal and general anesthesia on glucose and hemodynamic changes in patients undergoing curettage surgery.

## 3. Patients and Methods

This randomized clinical trial was conducted between 2012-2013 on patients aged 18 to 40 years, who reffered to Ahvaz Razi Hospital, Iran, and candidated for curettage surgery due to therapeutic abortion and had class І and II of American Society of Anesthesiologists (ASA). Patients with cardiovascular diseases, diabetes, endocrine disorders, chronic use of drugs, alcohol and cortisone and previous history of anxiety or stress were excluded from the study. After obtaining permission from the ethics committee of Ahvaz Jundishapur University of Medical Sciences (Ajums.REC.1393.56) and written consent from all patients, 50 female patients were selected and randomly divided into two groups of general anesthesia (GA group) and spinal anesthesia (SA group), equally.

In this study, patients did not receive any anti-anxiety medication before entering to operating room, patients were NPO for 8 hours. Standard monitoring was used including pulse oximetry, pulse rate, noninvasive blood pressure (NIBP) monitoring and 3 lead electrocardiogram monitoring. All patients received 5 ml/kg Ringer’s saline was prescribed. In GA group, patients received midazolam 0.05 mg/kg, fentanyl 2 μg/Kg and propofol 2 mg/kg and N2O-O2 50% -50%. SA group patients received 50 mg Xylocaine 5% (Orion Pharma Co-Finland) using a 25 gauge spinal needle at L4-L5 level. After assuring of neuroaxial blockade (lack of sensory perception of needle tip sharpness at T10 dermatome). During the surgery, hemodynamic paramaters such as blood pressure, heart rate and percentage of the O_2_ saturation were monitored continuously.

In both groups heart rate and mean arterial pressure (MAP) were measured at seven times (10 minutes before and 10, 20, 30, 40, 50, and 60 minutes after the initiation of anesthesia). Also, blood sugar was measured three times (10 minutes before and 20 and 60 minutes after initiation of anesthesia) by the index finger of the right hand and maintaining sterility conditions using glucometer (IME-DC-Germany).

### 3.1. Statistical Analysis

The sample size was analyzed by taking into account the power of 90% and a coefficient level of 95% based on analysis of variance for repeated measurements using NCSS software, calculated 14 samples, which for geeting coherence results we considered 25 samples in each group. t-test to compare blood glucose differences between two groups at any time and LSD multiple comparison test to compare blood sugar before, during and after surgery in both groups. All data were reported in terms of mean ± Standard Deviation (SD). Data were considered significant at the level of P < 0.05

## 4. Results

In this study, 50 female patients were divided into two anesthesia groups of the spinal group (SA group) and general group (GA group). None of the patients were excluded from the study. According to Table 1, there were not statistically significant differences between two groups in terms of demographic characteristics (age, height, and weight) and duration of anesthesia (P > 0.05).

**Table 1.** The Demographic Characteristics and Duration of the Anesthesia in Both Groups ^a^

Variables	GA group	SA group	P Value
**Age, y**	32.8 ± 1.77	31.7 ± 1.77	0.751
**Weight, kg**	62.6 ± 3.82	59.9 ± 4.29	0.631
**Height, cm**	158.7 ± 2.3	158.6 ± 2.87	0.956
**Duration of anesthesia, min**	25.2 ± 3.4	23.7 ± 5.59	0.719

^a^ Values are presented as mean ± SD (P > 0.05).

Based on Table 2, blood glucose levels of GA and SA groups show no significant difference at 10 minutes before, 20 and 60 minutes after initiation of anesthesia

**Table 2.** Blood Sugars of Patients During 10 Minutes Before, and 20 and 60 Minutes After Initiation of Anesthesia in Both Groups ^a^

Blood sugar	GA group	SA group	P Value
**10 min before anesthesia**	86.96 ± 13.32	88.60 ± 16.98	0.706
**20 min after anesthesia**	80.88 ± 13.13	85.88 ± 15.05	0.268
**60 min after anesthesia**	83.24 ± 15.78	90.04 ± 16.26	0.140

^a^ Values are presented as mean ± SD (P > 0.05).

According to Table 3, the MAP changes were 5,4% and 8% lower than baseline in GA group and SA group respectively (base line of MAP in GA and SA group was 106.7 ± 6.4 and 103.1 ± 8.1 respectively).

**Table 3.** Changes in MAP at Various Specified Timings in Two Groups

MAP	GA group	SA group	P Value
**10 min before anesthesia**	106.7 ± 6.4	103.1 ± 8.1	0.578
**10 min after anesthesia**	104.7 ± 5.3	111.8 ± 6.9	0.301
**20 min after anesthesia**	100.2 ± 1.5	108.6 ± 3.7	0.411
**30 min after anesthesia**	101 ± 4.5	106.8 ± 5.6	0.541
**40 min after anesthesia**	103.9 ± 7.2	108.4 ± 4.9	0.437
**50 min after anesthesia**	101.7 ± 4.8	100.3 ± 2.3	0.837
**60 min after anesthesia**	100.6 ± 2.3	101.6 ± 8.4	0.864

According to Table 4, heart rate changes in the GA group were maximum 12.5% lower than baseline, and in the SA group were maximum 14.5% lower than baseline.(base line of HR in GA and SA group was 95.3 ± 10.5 and 93.7 ± 7.3 respectively).

**Table 4.** Changes in Heart Rate at Various Specified Timings in Two Groups

HR	GA group	SA group	P Value
**10 Min before anesthesia**	95.3 ± 10.5	93.7 ± 7.3	0.729
**10 Min after anesthesia**	86.8 ± 9.8	109.5 ± 6.6	0.087
**20 Min after anesthesia**	83.4 ± 6.7	106.6 ± 8.4	0.091
**30 Min after anesthesia**	84.5 ± 8.2	99.8 ± 5.9	0.143
**40 Min after anesthesia**	88 ± 3.7	101.4 ± 2.1	0.234
**50 Min after anesthesia**	91.6 ± 8.3	95.2 ± 7.5	0.608
**60 Min after anesthesia**	96.1 ± 9.1	90.6 ± 8.7	0.712

## 5. Discussion

Based on results while the average blood glucose after the initiation of the anesthesia in GA group was lower than the SA group but no statistically significant differences was found between two groups. Furthermore, changes in the mean arterial pressure (MAP) and heart rate (HR) compared to baseline in the minutes of study in the GA group were lower than SA group. In one study, Moller et al. showed that patients undergoing abdominal hysterectomy with spinal or general anesthesia had a clear increase in plasma cortical and glucose levels during and after surgery (9).

Similarly Huiku et al. indicated that breast and gynecological surgeries led to hemodynamic changes during surgical incision. In this study, hemodynamic variables were higher than baseline after initiation of the surgery (10).

In general, blood glucose concentration will increase after surgery. Cortisol and catecholamines increase glucose production via increasing gluconeogenesis as well as reducing the peripheral glucose consumption. Similarly, the common mechanisms for stabilizing blood sugar are ineffective during surgery (5).

In clinical practice, these activities cause changes in heart rate and blood pressure and adrenaline biochemical fluctuations, noradrenaline and dopamine. Also type of surgery and anesthesia are involved in surgical stress (5).

In a similar study Ilies et al. showed that hemodynamic variations such as heart rate and systolic blood pressure in the general anesthesia group were less than spinal anesthesia group in elective urology and orthopedic surgeries (11). We obtained similar results.

Unlike our study, Moller et al.’s study showed that levels of plasma cortisol and glucose in the patients undergoing abdominal hysterectomy in the spinal anesthesia group were clearly lower than general anesthesia group during and immediately after the surgery (9). Our study found the opposite results.

One of the reasons that justify the difference between our results compared with above mentioned studies is experiencing psychological stress prior to surgery. Female gender considered as a risk factor for stress during operation (12). Lower blood sugar levels in the sedation group comparing with the spinal group is acceptable, possibly due to more mental stress, sympathetic nervous system activity and release of catabolic hormones in patients undergoing spinal anesthesia, who are awake. Also, lower level block in surgical procedures such as curettage can’t completely suppress the hormonal and metabolic changes (13). These procedures really takes a very small time interval and physicians usually do it by using general anesthesia, intravenous sedation or local anesthesia (14).

One of the limitations of this study was lack of measurement of serum stress hormones levels such as cortisol as well as blood sugar and lack of evaluation of Bispectral index (BIS) for assessment of patients with general anesthesia. Also, personality type difference and its effect on the stress response due to the surgery has not been evaluated. However, the positive aspect of this study was the simultaneous investigation of hemodynamic changes and blood sugar levels as surgical stress responses in patients undergoing curettage. Based on limitation and availability of limited datato assess the benefits and priorities of anesthesia techniques, further studies are recommended (15).

Blood glucose and hemodynamic changes caused by surgical stress were not significantly different between two groups.
